# The contribution of gender-based violence and network trauma to gender differences in Post-Traumatic Stress Disorder

**DOI:** 10.1371/journal.pone.0171879

**Published:** 2017-02-16

**Authors:** Derrick Silove, Jess R. Baker, Mohammed Mohsin, Maree Teesson, Mark Creamer, Meaghan O'Donnell, David Forbes, Natacha Carragher, Tim Slade, Katherine Mills, Richard Bryant, Alexander McFarlane, Zachary Steel, Kim Felmingham, Susan Rees

**Affiliations:** 1 Psychiatry Research and Teaching Unit, University of New South Wales, Sydney, Australia; 2 School of Psychiatry, University of New South Wales, Sydney, Australia; 3 Office of Medical Education, University of New South Wales, Sydney, Australia; 4 Department of Psychiatry, University of Melbourne, Melbourne, Australia; 5 School of Psychology, University of New South Wales, Sydney, Australia; 6 Centre for traumatic Stress Studies, University of Adelaide, Adelaide, Australia; Radboud University Medical Centre, NETHERLANDS

## Abstract

**Background:**

Posttraumatic stress disorder (PTSD) occurs twice as commonly amongst women as men. Two common domains of trauma, network trauma and gender based violence (GBV), may contribute to this gender difference in PTSD rates. We examined data from a nationally representative sample of the Australian population to clarify the characteristics of these two trauma domains in their contributions to PTSD rates in men and women.

**Methods:**

We drew on data from the 2007 Australian National Survey of Mental Health and Well-being to assess gender differences across a comprehensive range of trauma domains, including (1) prevalence of lifetime exposure; (2) identification of an index trauma or DSM-IV Criterion A event; and (3) the likelihood of developing full DSM-IV PTSD symptoms once an index trauma was identified.

**Results:**

Men reported more traumatic events (TEs) overall but women reported twice the prevalence of lifetime PTSD (women, 13.4%; men, 6.3%). Women reported a threefold higher level of exposure to GBV and were seven times more likely to nominate GBV as the index trauma as compared to men. Women were twice more likely than men to identify a network trauma as the index trauma and more likely to meet full PTSD symptoms in relation to that event (women, 20.6%; men, 14.6%).

**Conclusion:**

Women are more likely to identify GBV and network trauma as an index trauma. Women’s far greater exposure to GBV contributes to their higher prevalence of PTSD. Women are markedly more likely to develop PTSD when network trauma is identified as the index trauma. Preventing exposure to GBV and providing timely interventions for acute psychological reactions following network trauma may assist in reducing PTSD rates amongst women.

## Introduction

Posttraumatic stress disorder (PTSD) is a common and disabling mental disorder that incurs substantial social and economic costs to societies worldwide [1–5]. Criterion A of the fourth edition of the Diagnostic and Statistical Manual of the American Psychiatric Association (DSM-IV) defines PTSD as a reaction to an event that is threatening to the life or physical integrity of the self or close others, and to which the survivor exhibits an acute psychological response (horror, fear, helplessness) [6]. Once Criterion A is met, a full diagnosis of PTSD requires that the person manifests three domains of symptoms including the re-experiencing of trauma memories, for example as flashbacks and nightmares; avoidance of reminders of the event and emotional numbing; and heightened physiological arousal and reactivity. Symptoms must persist for at least a month and cause significant psychosocial dysfunction. Although the criteria for PTSD have been expanded in the most recent edition of the DSM (DSM-5), the symptom domains of DSM-IV applied in the present study continue to be regarded as core to the disorder [7, 8].

A consistent finding in the research literature on PTSD is that women exhibit twice the rate of the disorder as men, in spite of men experiencing greater lifetime exposure to traumatic events (TEs) overall [3, 9–12]. Clarifying the reasons for this gender disparity in PTSD rates may assist in furthering understanding of the pathogenesis of the disorder as well as in guiding the tailoring of interventions to suit the specific needs of men and women [13, 14].

Contention persists, however, concerning the reasons for the observed gender disparity in PTSD rates, the chief explanations offered being that women have an increased susceptibility to develop this response after trauma exposure and that women are differentially exposed to certain types of trauma that are particularly potent triggers of the disorder [13, 15]. In support of the first argument, a meta-analysis of 290 studies concluded that women were more likely than men to develop PTSD independent of the type of precipitating trauma, suggesting a general female susceptibility to more severe psychological reactions when confronted by TEs [16]. However, that finding should not obscure the possibility that women are more frequently exposed to trauma domains that are more potent in provoking PTSD, particularly in civilian populations not directly exposed to warfare. Gender based violence (GBV), constituting rape, other forms of gendered/sexual assault, intimate partner violence and stalking, represents a domain that may account in part for the gender disparity in PTSD. GBV exposure is far more common amongst women than men [17]; and the constituent abuses tend to be associated with a range of adverse personal and social reactions including stigma, shame and self-blame.

Remarkably, the research literature to date has not discriminated clearly between GBV and other forms of violence in determining the gender discrepancy in PTSD rates. Other forms of violence (including crime and non-gendered forms of assault involving acquaintances or strangers outside the home) are more commonly experienced by men [5, 16], in stark contrast to GBV where the gender pattern is reversed. In addition, other forms of violence are not as strongly associated with the adverse personal and social consequences (self-blame, stigma, guilt) so closely associated with GBV [2, 14, 18–24]. For these reasons, it is vital to disaggregate GBV and other forms of violence in attempting to define more clearly the source of gender differences in PTSD rates.

Network trauma is an additional domain that warrants attention in attempts to clarify the reasons for the gender difference in PTSD rates [25, 26]. Network trauma includes unanticipated illness, death or injury involving close others, assessed herein in accordance with DSM-IV. We note in parenthesis, however, that DSM-5 has narrowed the definition of this domain to learning of a violent or accidental event involving a close family member or friend [7]. Network trauma is common in both men and women, but evidence suggests that women exhibit a stronger immediate emotional response to these types of events [27, 28]; for example, women appear to have a greater tendency to develop PTSD after learning about a trauma involving close others [17, 29]. A study of a representative national sample may clarify further, however, the points in the chain (involving trauma exposure, the immediate response, and the development of PTSD) at which network trauma contributes to the gender difference in the prevalence of the disorder.

In the present analysis, we draw on data from the 2007 Australian National Survey of Mental Health and Well-being to examine what contributions GBV and network trauma make to the higher lifetime prevalence of PTSD amongst women. In relation to GBV, we hypothesized that greater environmental exposure to that trauma domain amongst women would make a substantial contribution to the gender disparity in PTSD. In relation to network trauma, we hypothesized that women were more likely to identify events from that domain as the index trauma or Criterion A of PTSD; and that they would have a greater propensity to manifest full symptoms of the PTSD after identifying such an event as the index trauma.

## Methods

### Data collection

The analysis was conducted on an existing dataset collected and held by the Australian Bureau of Statistics (ABS), the resource being accessible to researchers in Australia on application to the agency. As the official Australian Government population research agency, the ABS operates according to statutory provisions that ensure rigorous ethical review and conduct of all research, including adherence to strict procedures of voluntary recruitment, confidentiality, and obtaining informed written consent from participants [30]. The stringent ethical procedures implemented by the ABS exempt researchers from submitting proposed analyses of relevant ABS databases to university Human Research Ethics Committees (the equivalent of Institutional Review Boards).

The methodology, sampling and measures applied in conducting the second Australian National Survey of Mental Health and Well-being (2007) have been fully described elsewhere [31, 32]. The study involved a random, stratified, multistage, area probability survey, including persons aged 16 to 85 years drawn from the entire Australian population. Random selection of one person from 14,805 households without replacement for refusals yielded a total of 8,841 participants, including 4,027 (45.5%) men and 4,814 (54.5%) women; a response rate of 60%. The present analysis was applied to a weighted sample of 4,390 (49.7%) men and 4,451 (50.3%) women. Given that the sample may have differed to the whole base population at a national level, the ABS calculated and provided 60 replicate weights (including for age and gender) to standardize the sample according to the national structure on key socio-demographic variables. We therefore calculated the weighted estimates for each of the items (as provided by the ABS). ABS trained interviewers conducted face-to-face interviews in participants’ homes [32]. A follow-up study of non-responders indicated that the response rate did not introduce major biases in relation to the major indices assessed [33].

### Assessment of trauma events and PTSD

We followed a stepwise procedure to assess for gender differences: 1) in exposure to 29 lifetime TEs, and their aggregated thematic domains (see Table 1); 2) in nominating a TE as an “index trauma” (the method for deriving Criterion A or the worst trauma which leads onto an inquiry into PTSD symptoms, see hereunder); and 3) the likelihood of developing full PTSD symptoms after identifying an index trauma.

**Table 1:** Lifetime traumatic events (weighted) experienced by men and women (noting that individuals could nominate multiple traumatic events).

	Men (n = 4390)		Women (n = 4451)	
	Number	%	Number	%
**Accidents and Natural Disasters**				
Toxic chemical exposure	393	9.0	98	2.2
Life threatening automobile accident	746	17.0	392	8.8
Life threatening accident including on the job	444	10.1	127	2.9
Natural disaster	433	9.9	326	7.3
Man-made disaster	266	6.1	145	3.3
**Witnessing Trauma**				
Witnessed death/dead body or seriously hurt	1557	35.5	806	18.1
Saw atrocities	244	5.6	75	1.7
**War Events**				
Combat experience	167	3.8	5	0.1
Peacekeeper or relief worker	64	1.5	10	0.2
Civilian in war zone	182	4.1	142	3.2
Civilian in region of terror	158	3.6	103	2.3
Refugee	69	1.6	56	1.3
Purposely injured, tortured or killed someone	94	2.1	11	0.3
**Other Physical Violence**				
Beaten up by someone else	476	10.9	119	2.7
Mugged or threatened with a weapon	777	17.7	325	7.3
Kidnapped or held captive	34	0.8	45	1.0
**Non-genderual physical violence in early life**				
Beaten up by caregiver	242	5.5	214	4.8
Witnessed physical fight at home	394	9.0	504	11.3
**Other Trauma**				
Accidentally caused injury/death of another person	91	2.1	27	0.6
Life threatening illness	566	12.9	528	11.9
Life threatening related to death/injury	101	2.3	128	2.9
Some other event	215	4.9	238	5.4
Private event	180	4.1	263	5.9
**Gender based violence**				
Rape	89	2.0	406	9.1
Other sexual assault	211	4.8	698	15.7
Stalking	176	4.0	497	11.2
Beaten up by spouse/ partner	79	1.8	393	8.8
**Network Trauma**				
Unexpected death of loved one	1491	34.0	1635	36.7
Child with serious illness	297	6.8	399	9.0
Traumatic event to loved one	322	7.3	403	9.1
***Number of total Traumatic events***				
None	1042	23.7	1138	25.6
***At least one traumatic event***	3348	76.3	3313	74.4

### Step 1: Exposure to Traumatic Events (TEs)

All participants were asked whether they had been exposed at any point in their lives to one or more of a list of 29 TEs (yes/no), based on standard events used across countries participating in the World Health Organization World Mental Health Surveys [1]. Following past convention, we grouped the 29 TEs into thematic domains, except that we divided Other Physical Assault and GBV into separate categories [34]. The domains therefore included: (1) Accidents and Natural Disasters; (2) Other Physical Assault; (3) Exposure to Non-gender Violence in Early Life; (4) Witnessing Violence; (5) Network Trauma (involving traumatic losses and deaths); (6) War (including mass conflict); (7) Gender-based Violence (rape, other sexual assault, stalking, physical intimate partner violence); and (8) Other Trauma, including personal trauma that the respondent did not wish to specify (see Table 1).

### Step 2: Identifying an index trauma

Participants who recorded two or more of the 29 TEs were asked to identify one as their lifetime index trauma, defined as the event that stood out in their history as generating high levels of acute distress in the immediate aftermath, reflected in symptoms such as upsetting memories or dreams, feeling emotionally distant or depressed, experiencing trouble sleeping or concentrating, and/or feeling jumpy or easily startled (for participants who had only experienced one trauma type, this was taken to be their index event if they also endorsed having the required immediate psychological reaction to that occurrence). Only those participants who reported at least one TE and fulfilled the criterion of an index trauma (DSM-IV Criterion A) proceeded to a systematic inquiry about the presence of lifetime DSM-IV PTSD symptoms.

### Step 3: Assessing lifetime DSM-IV PTSD

Those with an index trauma were assessed for lifetime symptoms of PTSD (criterion B-D according to DSM-IV) using the module of the World Health Organization’s Composite International Diagnostic Interview (WMH-CIDI) version 3 [35]; the most globally applied measure of common mental disorders in contemporary national mental health surveys.

### Statistical analyses

#### Step 1: Exposure to Traumatic Events (TEs)

TEs experienced by men and women for each trauma domain are presented in Table 1. The percentages reported reflect the number of TEs (weighted) for men and women within each TE domain divided by the total number of men and women in the survey. Given that individuals could report multiple lifetime TEs, the percentages for each domain do not add up to 100%. The proportion test compared the percentage of men and women who endorsed a TE (or trauma domain), thereby providing an index of the gender difference in exposure.

#### Step 2: Identifying an index trauma

The number of index traumas nominated by men and women were documented for each trauma domain. The percentage reflected the number of index traumas for men and women within each domain, divided by the total number of men and women who reported any TE from that domain (noting that each individual could only name one index trauma, and that not all individuals who reported a TE identified an index trauma because not all individuals reported the level of immediate psychological reactivity required by DSM-IV Criterion A). The proportion test compared gender differences in identification of an index trauma according to each trauma domain.

#### Step 3: Assessing lifetime PTSD

The number of men and women who met DSM-IV criteria for PTSD based on their index trauma is reported for each trauma domain. The percentage reported for this index reflects the prevalence of PTSD for men and women within each trauma domain (and overall) divided by the total number of men and women who identified an index trauma, respectively. Prevalence ratios (PR) with 95% confidence intervals (95% CI) indicate gender differences in PTSD rates per trauma domain (and overall) [36]. In all instances, the reference category is men; PRs >1.00 indicate a higher rate of PTSD for women as compared to men and PRs< 1.00 indicate a higher rate of PTSD for men as compared to women. Significance levels are reported at p<0.05. All analyses were undertaken in SAS V9.3 (SAS Institute Inc., Cary, NC, USA, 2002–2010).

## Results

### Step 1: Exposure to Traumatic Events (TEs)

Table 2, indicates that men reported higher overall rates of TEs than women (women, n = 3348, 76.3%; men, n = 3313, 74.4%, p<0.05). Men reported greater exposure than women to the TE domains of accidents and natural disasters; witnessing trauma; war; and other physical assault (all p<0.01). Women reported significantly higher exposure to non-gender-related physical violence in early life than men (p<0.05).

**Table 2:** Prevalence of Traumatic Events (TEs), index trauma (index trauma); and lifetime Post-Traumatic Stress Disorder (PTSD) for men and women (weighted data).

Trauma domain (single/multiple events counted)	STEP 1: Prevalence of lifetime trauma events by trauma domain and sex^1^: n (%)		STEP 2: Prevalence of nominated index trauma amongst those reporting at least one lifetime TE by trauma domain and Sex^2^: n (%)		STEP 3: Prevalence of PTSD by lifetime trauma and index trauma: n (%)				Prevalence ratio of PTSD (reference men) in relation to index trauma domain (95% CI)
					PTSD in relation to lifetime trauma^1^		PTSD in relation to index trauma^2^		
	Men (n = 4390)	Women (n = 4451)	Men (n = 3348)	Women (n = 3313)	Men (n = 3348)	Women (n = 3313)	Men (n = 1068)	Women (n = 1702)	
Accidents and natural disasters	1572 (35.8)**	906 (20.3)	160 (4.8)**	**107 (3.2)**	127 (8.1)	128 (14.2)**	**15 (9.4)**	**16 (15.0)**	1.59 (0.8–3.1)
Witnessing trauma	1598 (36.4)**	831 (18.7)	150 (4.5)**	**77 (2.3)**	137 (8.6)	156 (18.8)**	**14 (9.3)**	**6 (7.8)**	0.83 (0.3–2.1)
War events	528 (12.0)**	266 (6.0)	62 (1.9)**	**17 (0.5)**	48 (9.1)	18 (6.9)	**18 (29.0)**	**2 (11.8)**	0.41 (0.1–1.6)
Other physical violence	1027 (23.4)**	426 (9.6)	90 (2.7)*	**63 (1.9)**	128 (12.4)	114 (26.8)**	**22 (24.4)**	**17 (27.0)**	1.10 (0.6–1.9)
Non-gendered physical violence in early life	501 (11.4)	584 (13.1)*	**71 (2.1)**	118 (3.6)**	76 (15.1)	134 (22.9)**	**25 (35.2)**	**34 (28.8)**	0.82 (0.5–1.3)
Other trauma	911 (20.8)	941 (21.1)	**144 (4.3)**	244 (7.4)**	103 (11.3)	165 (17.6)**	**44 (30.6)**	**61 (25.0)**	0.82 (0.6–1.1)
Gender based violence (GBV)	435 (9.9)	1313 (29.5)**	**62 (1.9)**	416 (12.6)**	82 (18.9)	320 (24.4)*	**26 (41.9)**	**187 (45.0)**	1.07 (0.8–1.5)
Network trauma	1782 (40.6)	2007 (45.1)**	**329 (9.8)**	660 (19.9)**	142 (8.0)	316 (15.7)**	**48 (14.6)**	136 (20.6)*	1.41 (1.1–1.9)*
**All traumatic events**	**3348 (76.3)**	3313 (74.4)*	**1068 (31.9)**	1702 (51.4)**	**212 (6.3)**	459 (13.9)**	**212 (19.9)**	459 (27.0)**	1.36 (1.2–1.6)*

^**1**^ **Lifetime traumatic events** (multiple events counted)

^**2**^**Lifetime index traumatic event** (single events counted only)

*Prevalence of traumatic events and of PTSD differed statistically by gender at p<0.05;

** PTSD differed statistically by gender at p<0.01;

**Accidents and Natural Disasters includes:** toxic chemical exposure, life threatening automobile and other accidents, natural/man-made disaster; **Witnessing Trauma:** witnessed death/dead body or someone seriously hurt, saw atrocities; **War Events:** combat experience, peacekeeper or relief worker, civilian in war zone, civilian in region of terror, refugee, purposely injured/tortured or killed someone; **Other Physical Violence:** beaten up, mugged or threatened with a weapon, kidnapped or held captive; **Non-gender physical violence in early life:** Beaten up by caregiver, Witnessed physical fight at home; **Other Trauma:** accidentally caused injury/death of another person, life threatening illness, some other event, private event; **Gender based violence (GBV):** rape, genderually assault, stalking, beaten up by spouse/ partner; **Network Trauma:** unexpected death of loved one, child with serious illness, traumatic event to loved one; ***Formula for 95% CI of Prevalence ratio (PR)*:** *log*_*e*_ *(PR)± 1*.*96 SE* where *SE ≈ √ [(1-p*_*1*_*)/n*_*1*_*p*_*1*_ *+ (1-p*_*2*_*)/(n*_*2*_ *p*_*2*_*)][36]*.

In relation to the two trauma domains of interest, both genders reported high levels of exposure to network trauma, although rates were statistically greater for women, p<0.01.

Forty five per cent of women (n = 2007 out of the total of 4451) reported network trauma; whilst the network trauma count for men (n = 1782 of a total of 4390) was 40.6%. Women reported a threefold higher rate of exposure to GBV than men (p<0.01). Specifically, 29.5% of women reported GBV (n = 1313) compared to 9.9% of men (n = 435).

### Step 2: Identifying an index trauma

Table 2 indicates that women identified more index traumas than men (women, n = 1702, 51.4%; men, n = 1068, 31.9%; p<0.01). In addition, there were distinctive patterns in reporting index traumas for men and women. Men identified proportionally more index traumas than women for the TE domains of accidents and disasters, witnessing trauma, other physical assault, and war trauma (all p<0.05). Women reported proportionately more index traumas than men for non-gender-related physical violence in early life (p<0.01). In addition, there was a two-fold difference between men and women in recording network trauma, p<0.01; the number of women experiencing that TE domain (n = 660 of 3313) was 19.9%; in comparison, for men, the percentage was 9.8% (n = 329 of 3348).

There was a seven-fold greater reporting of GBV as an index trauma amongst women compared to men (p <.001. The number of women who identified GBV (n = 416 of 3313 reporting any lifetime TE) was 12.6%; in comparison, the percentage of men reporting GBV was 1.9% (n = 62 of the 3348 reporting any TE).

### Step 3: TE domains and lifetime PTSD

Women showed a greater overall tendency to develop full PTSD symptoms than men following TE exposure: PR = 1.36; 95% CIs = 1.2–1.6(see Table 2). Specifically, the rate of PTSD for women (n = 459 of 1702 who identified an index trauma) was 27.0%, compared to 19.9% for men (n = 212 of 1068). However, of the individual TE domains, only network trauma showed a statistical gender difference (PR = 1.41, 95% CIs = 1.1–1.9), underscoring the role of that domain in the overall gender difference in the susceptibility to develop full PTSD symptoms once an index trauma was identified. In contrast, when GBV was the index trauma, men and women were equally likely to meet full symptom criteria for PTSD (PR = 1.07, CIs = 0.8–1.5).

## Discussion

Our findings, based on a representative sample of the Australian national population, are consistent with those from other countries [1, 20] in revealing that men reported greater exposure to TEs overall but women reported a two-fold higher rate of lifetime PTSD. When considered in relation to all TE domains, women had a greater overall propensity to identify a TE as an index trauma, the entry point for assessing a PTSD diagnosis; in addition, women had a higher likelihood of meeting full symptom criteria for PTSD once an index trauma was nominated. The traumas that men experienced more frequently, including interpersonal assault and violence, exposure to war (mostly attributable to military personnel deployed to conflict zones in other countries), and accidents, had a relatively low potency in generating PTSD. In contrast, the trauma domains that were more common amongst women, network trauma and GBV, each made a major contribution to PTSD. Compared to men, women reported a threefold greater exposure to GBV and a seven-fold increase in nominating a TE from that domain as the index trauma. There was no difference, however, between men and women in the likelihood of developing full symptoms of PTSD once a GBV event was nominated as an index trauma. These findings suggest that greater exposure to GBV and a tendency to nominate one of the constituent TEs as the index trauma both contribute to the gender difference in PTSD prevalence.

Network trauma was a common experience for both women and men, although the former recorded statistically greater levels of exposure. However, compared to men, women were more likely to identify a network trauma as the index trauma, a pattern in common with GBV. In contrast to GBV, however, women were more likely than men to develop full PTSD symptoms after nominating network trauma as the index trauma. Compared to GBV therefore, network trauma contributed to the gender disparity in PTSD in two ways; they were more likely to exhibit an intense immediate psychological reaction to the event, qualifying the TE for an index trauma; and they were more likely to develop full PTSD symptoms in the aftermath.

The strength of the study is the use of data from a large, nationally representative sample of men and women. The response rate was in the mid-range of comparable national mental health studies undertaken around the world [35]. A follow-up study confirmed that non-participants did not differ substantially from those interviewed in relation to the key indices assessed [37]. The CIDI-3 is the standard mental health diagnostic measure used in national surveys worldwide and diagnoses including PTSD have been validated against a gold standard clinical interview [38].

The cross-sectional design yielded prevalence data at one point in time, cautioning against inferring causal relationships, for example, between TEs and PTSD. In that regard, we note that the limited number of indices measured by the ABS precluded an examination of factors such as personal appraisal of events and more complex social and cultural influences which may influence the reporting of trauma and PTSD symptoms. In addition, we could not account for the duration of time between trauma exposure and onset of disorder, a factor that could influence the strength of the relationship. Finally, persons with disorders such as PTSD may over-report past trauma. Balancing against that concern, however, is the observation that there is less likely to be memory bias for the more severe traumas such as GBV[39].

The study was based on the DSM-IV criteria for PTSD, a classification system that was superseded by DSM-5 in 2013 [40]. The most important difference is that the tripartite symptom model of PTSD in DSM-IV (re-experiencing, avoidance/numbing, hyperarousal) has been expanded by the addition of a fourth domain in DSM5, that is, negative alterations in cognition and mood, a change that has led to an increase in overall number of symptoms and the redistribution of some of these across domains. Whether the results reported here will differ when a comparable analysis is conducted with the new system awaits further study. The items relating to GBV were restricted to severe physical abuse only and did not include psychological abuse that could add to mental distress [41, 42]. We restricted the analysis to categorical exposure to TEs (yes/no) because the range and frequency distributions of individual events varied greatly, and several TEs, such as domestic violence and stalking, tend to be ongoing or recurrent rather than limited to discrete events.

It is possible that gender itself influences the experience of trauma in addition to the objective differences between men and women in the range and frequency of TEs encountered. For example, gender may influence the selection of events that are nominated as the index trauma [29]. It is noteworthy, however, that both men and women identified network trauma as their most common index trauma, suggesting that systematic gender differences in the selection of trauma domains as the index trauma may not have exerted an undue influence on the nomination process.

There are substantive reasons why women nominate GBV as the index trauma. As has been often recognized, the callous disregard that perpetrators commonly exhibit for their victims' right to the sanctity of their own bodies [43] may have a particularly profound effect on the surviving woman’s psychological reaction to the event. Further, women’s claims of abuse may be met with disbelief or result in social stigma and even the misdirection of blame, family and communal responses that may intensify feelings of violation, isolation and alienation [44]. The general potency of GBV is attested to, however, by the finding that men and women have a high and equal likelihood of developing full PTSD symptoms after such abuses, consistent with findings of the first Australian National Mental Health Survey conducted 10 years earlier [45].

From a translational perspective, our findings underscore the importance of implementing primary prevention strategies aimed at protecting women from GBV at a society-wide and family level. From a policy perspective, reducing exposure to this form of abuse has the potential to make a major impact on the higher prevalence of PTSD observed amongst women. Clinically, it is important that, as part of the comprehensive care that survivors need, close monitoring is implemented to detect onset of PTSD in the aftermath of GBV [21].

Network trauma proved to be a high frequency event accounting for a substantial portion of PTSD cases overall, although exposure was statistically more common amongst women. Previous studies have implicated network trauma as a potent precipitant of PTSD in both men and women [25, 26]. For example, for both genders, sudden unexpected death of a loved one was the single most frequent cause of PTSD in an American sample of 2181 persons aged 18–45 years old [19]. In that study, however, PTSD was assessed with respect to a randomly selected trauma [25], as opposed to the present study which assessed PTSD with respect to a single index trauma from the list of TEs reported, an approach that is more aligned with clinical practice. The specific characteristics of network trauma in our study were that women were more likely to identify these events as the index trauma; and they had a greater propensity to develop full symptoms of PTSD once such a trauma was nominated. These findings suggest that network traumas have a distinctive psychological impact on women both in the acute and longer term period following exposure. Further research is needed to identify the factors that account for this susceptibility, including, amongst others, relevant biomarkers, and/or socially constructed factors related to women’s emotional attachment to the deceased, the timing and nature of traumatic losses, the gender-specific experiences of family and social disruptions, and the resources (material and interpersonal) available to women to cope in the aftermath [46, 47].

The data presented herein offer additional guidance in directing further research in this field. The evidence that women tend to be more emotionally involved in the lives of close others than men is supported by a national survey of over 20,000 Australians, in which women were found to be more personally affected by negative events in their partners’ lives than vice versa, women identifying strongly with the index experience as if it had occurred to them personally [48]. These observations are consistent with the general notion that the individual’s subjective appraisal of the meaning and impact of a TE is important to the risk of developing PTSD, a finding that requires further examination from a gender perspective [49].

Our findings offer guidance in shaping the social and psychotherapeutic response to experiences of traumatic network events. It may be that women require greater emotional and social support in the immediate period following such events. In relation to psychotherapeutic interventions for PTSD, focusing on the interpersonal impact of network traumas may be of particular relevance in the treatment offered to women [50, 51]. Our findings also raise questions whether events such as natural but sudden death or illness affecting a loved one should be excluded from consideration as a potential trigger of PTSD, a restriction now imposed in DSM-5. Although representing an attempt to remove normative experiences from the definition of a trauma, such a restriction may deter further examination of the potentially subtle differences in the nature, severity and impact of network events that distinguish men and women in their risk of developing PTSD.
